# Prevalence of non-communicable diseases among healthy male decontamination workers after the Fukushima nuclear disaster in Japan: an observational study

**DOI:** 10.1038/s41598-021-01244-z

**Published:** 2021-11-09

**Authors:** Toyoaki Sawano, Michio Murakami, Akihiko Ozaki, Yoshitaka Nishikawa, Aoi Fukuda, Tomoyoshi Oikawa, Masaharu Tsubokura

**Affiliations:** 1 Department of Surgery, Jyoban Hospital of Tokiwa Foundation, Iwaki, Fukushima Japan; 2 Department of Radiation Health Management, Fukushima Medical University School of Medicine, Fukushima, Fukushima Japan; 3 Research Center for Community Health, Minamisoma Municipal General Hospital, 54-6 Takamicho 2 chome, Haramachi, Minamisoma, Fukushima Japan; 4 Department of Health Risk Communication, Fukushima Medical University School of Medicine, Fukushima, Fukushima Japan; 5 Department of Breast Surgery, Jyoban Hospital of Tokiwa Foundation, Iwaki, Fukushima Japan; 6 Department of Internal Medicine, Soma Central Hospital, Soma, Fukushima Japan; 7 Department of Surgery, Sendai City Medical Center, Sendai, Miyagi Japan; 8 Department of Neurosurgery, Minamisoma Municipal General Hospital, Minamisoma, Fukushima Japan; 9 Present Address: Center for Infectious Disease Education and Research, Osaka University, Suita, Osaka Japan

**Keywords:** Health occupations, Medical research

## Abstract

The health status of healthy decontamination workers employed after the Fukushima nuclear disaster remains unclear. This study aimed to evaluate the prevalence of non-communicable diseases among such workers. In this observational study, questionnaires on lifestyle and social factors were administered as part of a health promotion program for decontamination workers in 2016 in Minamisoma City, Fukushima. The questionnaires and health check-up results were compared with those of the 2016 National Health and Nutrition Examination Survey (NHANES) in Japan. Overall, 123 male decontamination workers were enrolled; 93 (75.6%) were drinkers, and 84 (68.3%) were current smokers. The age-adjusted prevalence (95% confidence interval) of hypertension, dyslipidemia, diabetes mellitus, and obesity were 27.2% (20.1–34.4%), 30.4% (22.6–38.2%), 11.3% (5.5–17.1%), and 49.0% (39.0–58.9%), respectively. The age-adjusted prevalence in the NHANES were 32.8% (31.1–34.5%), 16.1% (14.5–17.6%), 7.0% (6.2–7.7%), and 31.2% (29.9–32.5%), respectively. The prevalence of obesity, dyslipidemia, binge drinking, and smoking were higher in healthy male decontamination workers than in the general population. Decontamination workers in disaster-struck areas may have higher risks of developing non-communicable diseases, possibly due to their original health status. Continuous monitoring of their health status and proper interventions are warranted.

## Introduction

Managing the health of vulnerable populations is an essential component of healthcare, given the wide disparity in access to healthcare services. Well-known examples of vulnerable populations in healthcare include the elderly and individuals with chronic diseases, disability, or a low socioeconomic status (SES)^1^. Previous research has highlighted the significant impact of social determinants on health outcomes^2^. In this regard, individuals with a low SES may have poorer access to required healthcare services, particularly in emergency settings such as natural disasters^3^. Furthermore, these vulnerable populations often experience difficulties in breaking the vicious circle of poverty and lose access to healthcare by themselves due to their socioeconomic factors^2^. Therefore, providing adequate support is critical for improving the access of vulnerable populations to healthcare in order to alleviate negative impacts on health in the aftermath of disasters.

Globally, occupational health and safety are regarded as major public health challenges in the medical field. The World Health Organization (WHO) has emphasized the need to protect vulnerable workers and manage their health^4^. For example, migrant workers have higher rates of adverse occupational exposure and health conditions owing to frequent changes in workplaces, and are therefore regarded as vulnerable workers^5, 6^. Low SES among migrant workers is a contributing factor to increased occupational health risks and reduced access to required health care services, resulting in higher risks of mortality associated with non-communicable diseases (NCDs)^7^. Notably, research on general and occupational health among migrant workers is limited.

Workers working at radioactivity-contaminated sites are considered migrant workers. Following the nuclear accident at the Fukushima Daiichi nuclear power plant (FDNPP) caused by the Great East-Japan Earthquake and tsunami on March 11, 2011, a class of migrant workers were engaged in work to mitigate the effects of radiation released by the accident^8, 9^. Decontamination processes near the power plant were commenced in an effort to minimize the level of external radiation to the general public following the accident. The number of decontamination workers in Fukushima Prefecture peaked in 2015 to approximately 30,000–40,000. Workers were employed from both Fukushima Prefecture and across the country^10^. Currently, the number of workers is decreasing annually, in parallel with the progress in decontamination in residential and agricultural areas in Fukushima Prefecture. Although detected individual radiation doses from external exposure among decontamination workers were low (averaging 0.6 mSv/year with a maximum of 7.8 mSv/year in 2015)^11^, a previous investigation suggested that decontamination workers had a higher risk of developing NCDs, which could also be related to their low SES^12^. A previous study documented that the prevalence of central obesity was higher in domestic migrant decontamination workers than in non-migrant workers^13^. Furthermore, our previous study suggested that of 113 decontamination workers admitted to the hospital, most were admitted with untreated NCDs^14^. However, to date, information on the health status of healthy decontamination workers is limited.

To address this gap in the literature, the primary aim was to determine the prevalence of NCDs among healthy decontamination workers, and the secondary aim was to compare the differences between the workers and the general population.

## Methods

### Study design, setting, and participants

**Figure 1:**
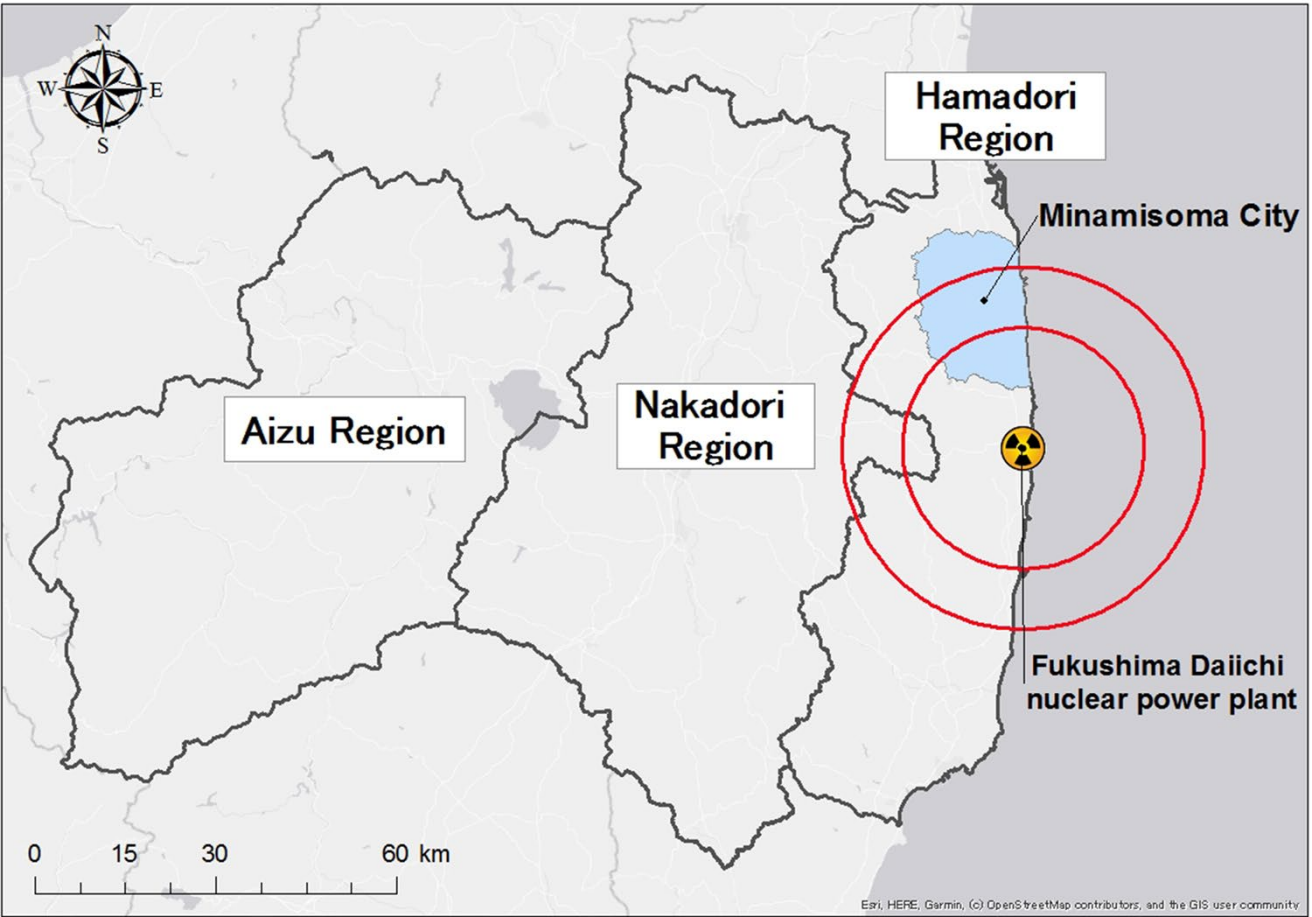
Location of Fukushima Daiichi nuclear power plant, Minamisoma City and regions of Fukushima Prefecture. Hamadori Region is a coastal area. Aizu Region is the most western inland region. Nakadori Region is located in the middle of these areas and is a city with prefectural government. Map data from © OpenStreetMap.

Due to the FDNPP accident that occurred on March 11, 2011, an extensive area of the Hamadori Region in Fukushima Prefecture was exposed to radiation contamination (Fig. 1). As a result, the Japanese government issued large-scale mandatory evacuation orders in consideration of the health risks of radiation exposure^9^. For the government to lift the evacuation order, the government and Ministry of Environment proposed a decontamination protocol to reduce the ambient dose equivalent in the contaminated areas^15^.

Following the accident at FDNPP, Minamisoma City in Fukushima Prefecture, located 13–38 km north of the power plant, was partially designated as an evacuation zone. As Minamisoma City was the closest area to the difficult-to-return zone around the FDNPP, many decontamination workers lodged in the city for transport convenience. From 2014, Minamisoma City experienced an upsurge of prefabricated houses for decontamination workers. Although the exact number of decontamination workers is unknown, approximately 10,000 unregistered workers were living in the city during the peak period.

Previous studies suggested that decontamination workers are exposed to a wide range of health risks^10^. Some of the plausible reasons for the poor lifestyle habits (excessive eating and drinking) among decontamination workers are fear of exposure to radiation, stress about radiation exposure risk, and the change in living environment. While the fear and stress related to radiation exposure are considered specific to the Fukushima accident, which was a radiation release disaster, environmental factors such as working alone are not limited to the Fukushima accident, and migrant workers all over Japan may be exposed to similar risks. In addition, the prevalence of NCDs may be higher among such migrant workers including decontamination workers after the FDNPP accident, due to their low SES.

NCDs are diseases that are not directly transmitted from person to person. NCDs are also called lifestyle-related diseases or chronic diseases. Most of these diseases are caused by one’s lifestyle habits and are preventable.

Our team offered health promotion activities for decontamination workers to promote their health via construction companies that employed these workers. However, a large proportion refused interventions, owing to the protection of personal information and privacy. Among various construction companies, Ishikawa Construction Co., Ltd, whose line of business includes decontamination work in Fukushima Prefecture, agreed to cooperate with our team to manage their employees’ health at the workplace and provide a health promotion program for decontamination workers.

This is an observational study that examined the baseline health status of decontamination workers using questionnaires and health check-up results in a health promotion intervention program for decontamination workers. After providing consent for participation in this study, participants were requested to complete a questionnaire, and they received a lecture session about the prevention of NCDs and interpretation of their check-up findings, which mainly included height, weight, blood pressure, as well as laboratory tests related to lifestyle. In the 20-min lecture, a medical doctor explained the mechanism of NCDs caused by unhealthy lifestyles and conveyed the horror of stroke and acute coronary syndrome, which is caused by worsening NCDs. In the session on interpreting the health check-up results, several doctors (of various specialties) reviewed the results of each participant's check-up findings and suggested how they should improve their lifestyle and whether they needed medical attention. However, the doctors did not perform any physical examinations. The participants were also offered opportunities to be consulted individually by the physicians.

Our study population included decontamination workers who were employed by Ishikawa Construction Co., Ltd. and participated in health promotion program that were held four times between February 20, 2016 and August 23, 2016 by our medical team in Minamisoma Municipal General Hospital. Of 124 decontamination workers included in the study, one female participant was excluded from this study, considering the potential confounding effects of sex differences on the data.

### Patient and public involvement

Since this study was a cross-sectional observational study, the participants were not involved in the research design and conduct of the study.

### Survey items

Our survey included the following items obtained from the questionnaire and health check-up findings:

1. Diseases diagnosed currently and previously by a physician.
2. Presence of NCDs such as hypertension, dyslipidemia, and diabetes mellitus (no patient with type 1 diabetes was identified, this study included only patients with type 2 diabetes), based on the medical examinations provided in our health program.
3. Demographic data such as (1) residential address, (2) past medical history and diseases being treated, (3) age, (4) height, (5) weight, (6) amount of alcohol consumed, (7) smoking status, (8) marital status, and (9) education level.

### Variables

In this study, the term ‘local worker’ referred to a decontamination worker whose residential address was in Hamadori and Nakadori regions of Fukushima Prefecture. (Fig. 1). In contrast, ‘migrant worker’ referred to workers from the Aizu Region of Fukushima Prefecture or from outside Fukushima Prefecture (Fig. 1). We counted the number of workers diagnosed (or responded in the questionnaire that they had been diagnosed) with hypertension, dyslipidemia, or (type 2) diabetes mellitus. We calculated the body mass index (BMI) of each participant and classified those with BMI ≥ 30 kg/m^2^ as obese and those with a BMI ≥ 25 kg/m^2^ as overweight, per the WHO definition^16^.

Regarding alcohol consumption categories, participants who reported consumption of any type of alcoholic beverage were referred to as drinkers. Those who self-reported consuming over 40 g of alcoholic beverages per day or more than 5 cups of sake equivalent per drinking session were classified as high-risk drinkers with increased risk of NCDs, according to the definition of the National Health and Nutrition Examination Survey (NHANES) in Japan^17^. With regards to smoking status, current smokers were identified; among participants who were regular current smokers, those who reported smoking more than 15 cigarettes per day were specifically noted. Regarding marital status, participants who currently had a marital partner were categorized as married. All unmarried participants were classified as single, regardless of whether they had never been married, divorced, or had a deceased spouse. Education level was used to evaluate the socioeconomic demographics among the decontamination workers. Education level was categorized into three levels: lower, ≤ 9 years, i.e., junior high school; middle, 10–12 years, i.e., high school and technical college; and higher, ≥ 13 years, i.e., university degree. All missing data were listed in the results section and excluded from the analysis.

### Statistical analysis

The chi-square test was used to investigate the differences in the prevalence of obesity and NCDs, including hypertension, dyslipidemia, and diabetes mellitus, between local and migrant workers. The prevalence of hypertension, dyslipidemia, diabetes mellitus, and obesity were compared between different education levels using the chi-square test. Age-adjusted prevalence and 95% confidence intervals (CIs) were calculated for each NCD in healthy male decontamination workers. Additionally, differences in the age-adjusted prevalence between this study and the NHANES were calculated. Data were compared with the NHANES 2016 data. Age adjustment was performed using the 1985 model in Japan^18^. In this analysis, four workers were excluded because the NHANES data only included individuals aged 20 years and above. One worker above 70 years of age was excluded from the analysis to remove outlier data.

### Ethical considerations

This research was approved by the ethics committees of Minamisoma Municipal General Hospital (Approval Number: 2-11) and Fukushima Medical University (Approval Number: 3065). All participants of this study were informed about the potential discomfort or adverse events they could experience while participating in this study, and they agreed to participate before completing the questionnaire. All participants provided informed consent for the study. The study was performed in accordance with the principles of the Helsinki declaration.

## Results

In total, 123 male participants were included in this study. Most of the participants were classified as ‘migrant workers’ (n = 100, 81.3%), because they had a certificate of residence at non-local addresses. The numbers of drinkers and current smokers were 93 (75.6%) and 84 (68.3%), respectively. Among the drinkers, 64 (52.0%) were considered to have a higher risk of developing NCDs. Regarding marital status, 65 (52.8%) were unmarried. Among the participants, 21 (17.1%) had an educational level below secondary school (Table 1).

**Table 1:** Baseline characteristics among 123 healthy male decontamination workers.

Characteristics		All patients (n = 123)
**Age, median (range), years**		48 (19–72 )
**Age distribution, years**		
≤ 19	4	3.3%
20–29	13	10.6%
30–39	18	14.6%
40–49	31	25.2%
50–59	24	19.5%
60–69	32	26.0%
≧70	1	0.8%
**Origin**		
Local†	23	18.7%
Other place	100	81.3%
**Alcohol**		
User	93	75.6%
High-risk drinker	64	52.0%
Non-responder	2	1.6%
**Smoking**		
Current smoker	84	68.3%
15 cigarettes/day or more	77	62.6%
Non-responder	1	0.8%
**Marital status**		
Unmarried††	65	52.8%
Married	53	43.1%
Non-responder	5	4.1%
Education level*		
Lower	21	17.1%
Middle	72	58.5%
Higher	28	22.8%
Non-responder	2	1.6%
**Body Mass Index (BMI)**		
Obesity (≥ 30.0 kg/m^2^)	14	11.4%
Overweight (≥ 25.0 kg/m^2^)	60	48.8%
**Health check-up results available**	106	86.2%

^†^ Locals were defined as workers who originated from Hamadori or Nakadori region in Fukushima Prefecture.

^††^ Persons who divorced or were never married.

*Education level was categorized into three levels: lower, ≤ 9 years, i.e., junior high school; middle, 10–12 years, i.e., high school and technical college; and higher, ≥ 13 years, i.e., university degree.

Among the participants, 60 (48.8%) were diagnosed as being overweight (BMI ≥ 25.0 kg/m^2^). Hypertension was found in 42 (34.1%), dyslipidemia in 42 (34.1%), and diabetes mellitus in 14 (11.4%) workers (Table 2). No statistically significant differences in the prevalence of obesity and NCDs were observed between local and migrant workers, between different marital statuses, and between different education levels (Table 2).

**Table 2:** Prevalence of non-communicable diseases among 123 healthy male decontamination workers.

	Hypertension		Dyslipidemia		Diabetes mellitus		Obesity	
Total (n = 123)	42	34.1%	42	34.1%	14	11.4%	60	48.8%
Local (n = 23)	11	47.8%	8	34.7%	2	8.7%	14	60.9%
Migrant (n = 100)	31	31.0%	34	34.0%	12	12.0%	46	46.00%
Pearson's chi squared test		p = 0.1249		p = 0.9431		p = 0.6103		p = 0.1983
Married (n = 65)	19	29.2%	20	30.8%	5	7.7%	30	46.1%
Unmarried (n = 53)	21	39.6%	22	41.5%	8	15.1%	29	54.7%
Pearson's chi squared test		p = 0.2355		p = 0.2255		p = 0.2015		p = 0.3548
Lower (n = 21)	8	38.0%	7	33.3%	3	14.3%	11	52.3%
Middle (n = 72)	26	36.1%	24	33.3%	5	6.9%	40	55.6%
Higher (n = 28)	8	28.6%	11	39.2%	6	21.4%	9	32.1%
Pearson's chi squared test		p = 0.7283		p = 0.8452		p = 0.1155		p = 0.1054

The age-adjusted prevalence of NCDs (hypertension, dyslipidemia, and diabetes mellitus) and obesity for decontamination workers aged between 20 and 60 years is presented in Table 3. The age-adjusted prevalence (95% CI) of hypertension, dyslipidemia, diabetes mellitus, and obesity among participants were 27.2% (20.1–34.4%), 30.4% (22.6–38.2%), 11.3% (5.5–17.1%), and 49.0% (39.0–58.9%), respectively. The age-adjusted national prevalence of hypertension, dyslipidemia, diabetes mellitus, and obesity in the NHANES were 32.8% (31.1–34.5%), 16.1% (14.5–17.6%), 7.0% (6.2–7.7%), and 31.2% (29.9–32.5%), respectively. The prevalence of dyslipidemia and obesity were significantly higher in decontamination workers than in the general population.

**Table 3:** Age adjusted prevalence of non-communicable diseases and obesity among the workers in their 20–60 (n = 118).†

Age	Total numbers	Hypertension	Dyslipidemia	Diabetes Mellitus	Obesity
20–29	13	1	0	0	5
30–39	18	2	7	4	10
40–49	31	7	11	2	16
50–59	24	15	11	4	14
60–69	32	16	12	4	12
Crude prevalence, %		34.7	34.7	11.9	48.3
Age-adjusted prevalence (95% CI), %††		27.2 (20.1–34.4)	30.4 (22.6–38.2)	11.3 (5.5–17.1)	49.0 (39.0–58.9)
Age-adjusted national prevalence (95% CI), % *††		32.8 (31.1–34.5)	16.1 (14.5–17.6)	7.0 (6.2–7.7)	31.2 (29.9–32.5)
ΔAge-adjusted prevalence (95% CI), %**		− 5.6 (− 12.9–1.8)	14.3 (6.4–22.2)	4.4 (− 1.5–10.2)	17.7 (7.7–27.8)

Confidence interval (CI).

^†^Four workers were excluded because the data of the National Health and Nutrition Examination Survey only included persons ≥ 20 years old. Only one worker, over the age of 70 years, was excluded from the analysis.

^††^Age adjustment was performed using the 1985 model in Japan.

*Based on the data from the National Health and Nutrition Examination Survey.

**Difference in age-adjusted prevalence between this study and National Health and Nutrition Examination Survey.

## Discussion

In this study, we investigated the NCD prevalence of 123 healthy male decontamination workers who participated in a company intervention-based health promotion program conducted by a local hospital in the disaster-struck area of the FDNPP accident. Notably, our study findings demonstrated that the age-adjusted prevalence of dyslipidemia and obesity were significantly higher among healthy male decontamination workers than among the national population. In contrast, the prevalence of hypertension and diabetes mellitus among decontamination workers were comparable to that of the general male population in Japan. No statistically significant differences were noted in the prevalence of NCDs and obesity between the class of workers (migrant versus local), marital status, and SES.

No evident differences were observed in the prevalence of NCDs and obesity, depending on the social background of decontamination workers, such as place of residence, marital status, and education level in this study. While our previous study indicated that the SES of decontamination workers as a whole may be low^14^, for education level, which is a major component of SES, 17.1% of participants had an education level of junior high school education or less, which was equivalent to that of the general Japanese population (18.8%) from the 2012 national census data^19^. Perhaps, it may be unlikely that there is no difference in health status depending on the social background of decontamination workers. Rather, this observation may be due to the possibility that the SES of the decontamination workers who participated in this study was relatively high, due to the small number of study participants or the specificity of the decontamination workers’ occupations. Other than the social factors among decontamination workers, it has been suggested that drastic changes in the living environment, such as diet and sleeping habits, may worsen the health status of migrant workers; however, these factors were not evaluated in this study due to the limited space in the questionnaires.

The social background of participants in this study was relatively favorable, compared with that of decontamination workers in general. Nevertheless, the prevalence of obesity and dyslipidemia and proportion of drinkers and smokers were significantly higher in the study population than in the general Japanese population. Furthermore, the proportion of harmful alcohol drinkers and current smokers among healthy male decontamination workers in this study (52.0% and 68.3%, respectively) was higher than that among the Japanese male general population reported in the 2016 NHANES (14.6% and 30.2%, respectively)^17^. These results are in accordance with those of previous studies, which have reported a high prevalence of high-risk drinking and smoking habits among hospitalized decontamination workers, whereas participants in this study had a significantly higher SES^14^. It is conceivable that other lifestyle choices such as diet may be poorer in decontamination workers than in the general public, thereby contributing to the development of NCDs.

Barriers currently exist in the implementation of health management interventions among workers with a low SES who are at risk of adverse health outcomes, such as decontamination workers. Our project initially aimed to implement a health promotion program and health interventions targeting a broad range of decontamination workers by recruiting companies involved in decontamination in the Fukushima Prefecture with the help of the local government. However, most companies involved in decontamination declined to participate in our research study due to concerns that it would expose the lack of proper health care management of their workers. In fact, there have been a case report that a company that hired decontamination workers concealed a work-related injury, perhaps for fear of exposing the poor health management of their own employees^20^. As a result, our survey may have only included a corporation with an adequate health and safety management system. Notably, the construction company that cooperated in this survey agreed to conduct a health promotion program for their employees, indicating superior healthcare management and safety practices relative to that of other companies. This may be reflected in the better environmental background of their employees. In sum, careful consideration is required when evaluating the general health profile of decontamination workers based on our study findings, as the health risks of the participants in our study may have been underestimated. Given the limited medical resources in disaster-affected areas, the poor health condition of decontamination workers moving into affected areas may place further strain on already-poor medical resources in the area. Further medical and health surveillance is required to assess the general health profile of decontamination workers and to promote the development of occupational health management systems.

## Limitations

To the best of our knowledge, this is the first study to investigate the health condition of healthy decontamination workers. Nevertheless, this study has several limitations. First, the results of this study may have been influenced by selection bias. This study was conducted with workers who agreed to participate in a health promotion program and were employed by construction companies that allowed their employees to participate in the program. Participants in this study may have been healthier and more health-conscious than other decontamination workers in the same area. If the data of all decontamination workers who received health check-ups at the hospital can be analyzed, this problem may be addressed in the future. Second, as health examination results were not available for some participants, several cases of lifestyle-related diseases may have been overlooked. Third, the sample size was too small, and most of the participants were migrant workers. Fourth, this was a cross-sectional study, and it was not possible to compare the drinking and smoking rates before and after the intervention or before and after the start of the decontamination process. In the future, it would be desirable to conduct research in a setting with more participants, after adjusting for the number of local and migrant workers.

## Conclusions

The prevalence of obesity and dyslipidemia were higher in healthy decontamination workers who participated in the health consultation program than in the general population, despite the relatively high SES of the study participants. Our results suggest that decontamination workers may be at a higher risk of developing NCDs, possibly due to their original health status and lifestyle habits, and continuous monitoring of their health status is warranted.

## Data Availability

Since the data set in this study contains personal information, it can be disclosed upon individual request, but cannot be made public.
